# Enzootic Bovine Leukosis in Italy: Epidemiological Issues after Free Status Recognition and Measures Applied to Tackle the Last Persistent Clusters

**DOI:** 10.3390/pathogens10111475

**Published:** 2021-11-12

**Authors:** Cecilia Righi, Carmen Iscaro, Stefano Petrini, Roberto Lomolino, Francesco Feliziani

**Affiliations:** 1 Istituto Zooprofilattico Sperimentale Umbria-Marche “Togo Rosati”, Via Gaetano Salvemini, 1, 06126 Perugia, Italy; c.righi@izsum.it (C.R.); s.petrini@izsum.it (S.P.); f.feliziani@izsum.it (F.F.); 2 Ministero della Salute, Viale Giorgio Ribotta, 5, 00144 Roma, Italy; r.lomolino@sanita.it

**Keywords:** cluster, enzootic bovine leukosis, eradication plan, gap analysis, Italy, surveillance plan

## Abstract

Enzootic Bovine Leukosis (EBL), caused by the bovine leukemia virus (BLV), has been eradicated in over 20 countries, most of which are in Western Europe. The European Commission, in 2017, declared Italy to be an officially EBL-free country by means of Commission Implementing Decision (EU) 2017/1910, despite the presence of some infection clusters located in four regions of Central-Southern Italy. As a consequence of persisting infection, the Italian Ministry of Health established specific eradication measures in these areas. In collaboration with the National Reference Laboratory for the Study of Ruminant Retroviral Infectious Diseases, the Ministry of Health employed data from the veterinary information system digital platform, combined with a gap analysis exercise, to monitor and verify the progress of control activities within infection clusters during the period 2018–2021. Our aim was to identify any remaining gaps and, consequently, specific measures to eliminate the factors favouring EBL persistence, on the basis of a description and analysis of the current data regarding epidemiological trends in Italian clusters. The final goal is to achieve the implementation of a less expensive surveillance plan in these areas, as well. The results of comprehensive analysis showed that the eradication activities had been effectively implemented by official local veterinary services, resulting in a drastic reduction of EBL outbreaks in most territories during the period 2018–2021.

## 1. Introduction

Enzootic Bovine Leukosis (EBL) [1,2] is a chronic infectious disease caused by the bovine leukaemia virus (BLV), an exogenous *Deltaretrovirus* belonging to the *Retroviridae* family.

EBL occurs mainly in adult cattle (*Bos taurus*), but other species are susceptible to experimental infection (e.g., sheep—*Ovis aries*) [3] and infection under natural conditions (e.g., water buffalo—*Bubalus bubalis*) [4,5]. Moreover, the potential risks of BLV for human health were recently confirmed, because the virus was detected in the breast tissue and blood of Iranian women using nested PCR [6], although there is no other robust evidence of human infection with EBL.

Usually, transmission is due to direct contact of mucosal surfaces or broken skin through a mixture of blood, exudates, and tissues [7]. Indeed, iatrogenic infection through blood-contaminated needles or surgical instruments [8] and transmission from dams to calves via ingestion of infected colostrum or milk [9] could represent other relevant routes of infection.

However, the spread of BLV is usually slow, and the virus can be easily inactivated by common disinfectants [9]. Vertical transmission via the placenta or by semen and the bites of blood-sucking insects (*Stomoxys calcitrans*) [10,11] probably account for a small proportion of cases [7].

Recent reports show that vertical transmission of BLV is more often found to be dependent on the dam’s viral load. Dams with higher BLV loads can be a source of maternal infection. About 50% of calves are positive after birth from such dams [12]. Intrauterine infection has also been proven [13]. Even in horizontal transmission, it has been suggested that high-viral-load cattle could be a potential spreader in the herd by phylogenetic analysis of *env* gene of BLV [14]. On the other hand, it has also been shown that cattle with low viral load are rarely an infection source [15].

Serological detection using the agar gel immunodiffusion (AGID) test or, mainly, by enzyme-linked immunosorbent assay (ELISA), is the most effective tool for controlling the spread of the infection and removing infected animals.

This viral disease is characterised by a very extended course, and 60% of infected cattle remain clinically silent in an aleukaemic state, while approximately 30% exhibit persistent lymphocytosis [16] and only 5–10% develop lymphoid tumours [17]. No vaccines or effective therapies are available for EBL.

Several reports have shown associations between the seroprevalence of EBL and milk production losses, reduced slaughter value, mortality and abortion, reproductive loss, and increased susceptibility to other diseases [18,19].

The disease has a strong economic impact, due to treatment costs (surveillance activities, veterinary services and facilities, medication costs, and extra farm-labour costs), annual losses to dairy producers and consumers [20], reduced export competitiveness, and increased consumer concerns regarding safety [18,19].

**Figure 1:**
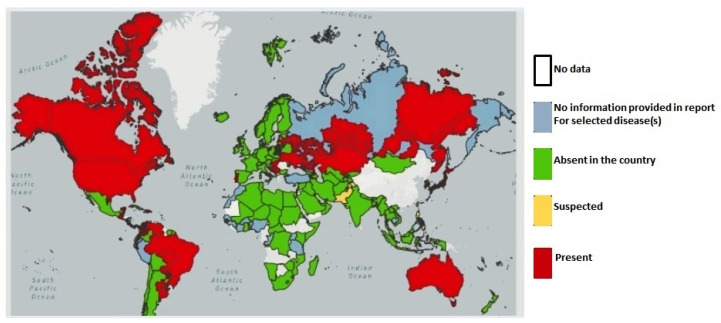
EBL outbreaks notified worldwide in 2021 [21].

EBL was first reported in Germany in 1874; then, through the trade of live animals, the disease expanded worldwide, and is currently present in some areas of Africa, America, Asia, Oceania, and Europe [21] (Figure 1).

**Figure 2:**
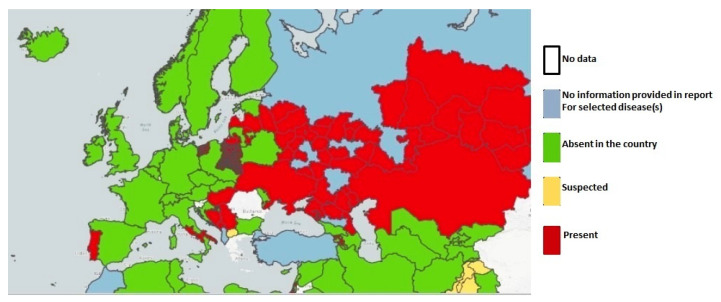
EBL outbreaks notified in Europe in 2021 [21].

Although several European Union (EU) Member States have obtained EBL-free status [22], some outbreaks are still present in France, Portugal, and the United Kingdom [23] (Figure 2).

The EU first enacted a specific legislation [24]; consequently, Italy adopted a mandatory eradication plan [25], by using the “test and removal” strategy for seropositive animals. When Italian regions achieved EBL-free status, it was necessary to implement a surveillance plan according to the legislative decree (D.Lgs) n.196, 22/05/1999 [26].

Currently, both regulations have been replaced by Commission Regulation (EU) 2016/429 [27]. EBL is a notifiable disease in European countries in compliance with Commission Implementing Regulation (EU) 2020/2002 [28]. It is subject to official control measures such as screening or monitoring precautions at borders, control of within-country movements, and stamping out [2].

In 2017, after application of the EBL eradication plan for more than 20 years, Italy was officially declared EBL free by Commission Implementing Decision (EU) 2017/1910 [29]. Therefore, all the Italian regions in which EBL was completely eradicated implemented a surveillance plan to prevent its reintroduction.

Nevertheless, some infection clusters persisted in restricted areas in four regions: Apulia, Campania, Latium, and Sicily, the latter until March 2021 [30,31]. In these territories, tailor-made eradication plans are in force, as well as special EBL control measures established by the Ordinanza Ministeriale (O.M.) on 28 May 2015 [32].

The Italian Reference Laboratory for the Study of Ruminant Retroviral Infectious Diseases (CEREL) is in charge of producing periodic reports to verify the progress of the EBL control activities. These reports are evaluated together with the Ministry of Health to manage any critical issues. The main available tool consists of the digital platform of the veterinary information system, called VETINFO [33], but recently other tools have been considered to evaluate the epidemiological situation and surveillance activities.

The purpose of this study was to describe and analyse the data on the EBL epidemiological trends of Italian clusters subjected to specific eradication plans, so as to identify possible remaining gaps and, consequently, specific measures to eliminate the factors favouring EBL persistence in these territories, in order to facilitate the attainment of freedom from the disease and allow the adoption of a less expensive surveillance plan.

## 2. Materials and Methods

### 2.1. European and National Legislation

In April 2021, Commission Regulation (EU) 2016/429 [27] on transmissible animal diseases, known as the “Animal Health Law” (AHL), was applied by the European Parliament and the Council to streamline the previously huge number of legal acts into a single law and to focus on key priorities: the prevention and eradication of disease. EBL is included in the list of animal diseases considered in Commission Regulation (EU) 2016/429 [27] and Commission Delegated Regulation (EU) 2018/1629 (Annex II) [34]. Moreover, in Commission Implementing Regulation (EU) 2018/1882 [35], EBL is included in a list of animal diseases relevant to EU intervention (category C) and is subject to optional eradication programs.

In this context, Commission Delegated Regulation (EU) 2020/689 [36] lays down ground rules for surveillance programs, disease control strategies, and measures to be applied in the case of suspected and confirmed infections; it establishes the criteria required to obtain and maintain official disease-free status, as well as the diagnostic methods to be used. Moreover, Commission Implementing Regulation (EU) 2021/620 [22] contains the updated list of all member states and regions officially recognised as EBL-free areas. These territories should implement individualised surveillance plans to maintain their official disease-free status.

In Italy, eradication efforts have been applied since 1996, as provided by the national eradication plan [25]. Once declared officially EBL-free, each region was allowed by the Health Ministry to adopt its own surveillance plans, as described in the legislative decree (D.Lgs) n.196, 22 May 1999 [26]. In any case, EBL legislation provides for the testing of all breeding farms by means of the serological investigation of all bovines (and buffalos) older than 12 months, twice a year, in order to achieve the eradication goal. Both the AGID (serum samples) and ELISA (serum or milk) tests are recommended by the World Organisation for Animal Health [2] for serological diagnosis of BLV infection, although ELISA is considered more sensitive and reliable, besides allowing the use of serum pools [31]. In particular, in four Italian infection clusters, official laboratories perform the ELISA test by serum matrix, and in the case of positive results, CEREL also carries out the AGID test.

A region is officially declared EBL free when all the cattle and buffalo farms in the region are examined annually, and the prevalence of BLV outbreaks is below the threshold of 0.2% (of controllable herds), according to EU regulatory criteria. In this case, the EBL-free region implements surveillance activities by testing all animals older than 24 months, according to a multi-annual plan (for instance, 20% of farms to be controlled each year for 5 years).

Following the recognition of the disease-free status of Italy in 2017 [29], the Italian Ministry of Health introduced general guidelines to standardise EBL surveillance activities in the 2018–2023 period at the national level. During this period, all Italian regions must carry out surveillance activities to provide evidence of the absence of EBL circulation.

Although the program has succeeded in eradicating EBL from most Italian regions, some areas, mainly located in southern regions such as Apulia, Campania, Latium, and Sicily, are lagging behind the northern regions [31].

As a consequence of persistent EBL infection, the Italian Ministry of Health, by means of Ministerial Ordinances (O.M.) 28 May 2015 [32] and O.M. 23 June 2020 [37], established specific additional EBL control measures within infection clusters, including electronic identification of the animals and registration in the National Database (BDN), monitoring of all the cattle farms twice a year by conventional serological screening of animals older than 6 months through blood sampling, and more severe measures in terms of biosecurity [38].

To define the EBL clusters, the Ordinance lists the following conditions: (1) level of herd prevalence > 0.2%; (2) persistence of the infection for at least 5 years; (3) epidemiological correlations between farms where EBL is circulating or persists; and (4) any other condition where measures provided by national legislation have been ineffective.

### 2.2. Data Source: VETINFO/Dashboard

A digital tool for the monitoring, management, and control of national eradication and surveillance plans for several diseases, such as EBL, called “Dashboard”, was devised and realised through the establishment of a designated Working Group (WG). Currently, it is available on the VETINFO platform, the national platform of veterinary information systems, and is accessible only through authentication.

The system allows verification of EBL epidemiological trends, surveillance and eradication activities, and compliance of the entire national territory with the current rules. Data can be displayed over the entire national territory, as well as at the regional and cluster levels. The system highlights the critical issues in persistent clusters of infection and the adoption of measures to overcome them.

The Dashboard collects data concerning the number of Italian cattle populations from the National Database (BDN), control activities of local veterinary services in breeding (National Veterinary System for Animal Health Activities Management, SANAN), notification of suspected EBL cases, and confirmed outbreaks (National Veterinary Information System on Animal Diseases, SIMAN). Moreover, it shows data about infected animals, timing of outbreak eradication, and use of pastures.

### 2.3. Gap Analysis Exercise

To monitor and improve the application of the EBL eradication plan within infection clusters, the Ministry of Health, in collaboration with CEREL, used a gap analysis approach. For this purpose, a qualitative survey was conducted in 2020 in four Italian infection clusters (in the regions of Apulia, Campania, Latium, and Sicily) to describe existing EBL eradication activities with the aim of identifying gaps and opportunities.

In particular, a questionnaire was designed for each cluster to collect information about:

- Current epidemiological situation and optimal standards to be achieved.
- Redefinition of the next objectives of the eradication plan.
- Identification of gaps between the current and the desired situation, and of the measures to be applied.
- Evaluation and identification of the resources needed to fill the gaps.
- Identification of factors that could promote or limit the performance of surveillance activities.

The questionnaire comprised five key sections about the implementation of the EBL eradication process: control of the local area and cattle/buffalo farms, control of animals, application of the planned sanitary measures, and effectiveness checks. A qualitative method for evaluating the gap analysis exercise and its results was selected on the basis of the following criteria:

A—Objective completely achieved.

B—Objective partially achieved.

C—Objective not achieved.

Moreover, a web conference between the referents of the Ministry of Health of the regions and CEREL was conducted to review the answers to the questionnaires and thus standardise the data collection. Finally, all data were entered into a report, which summarised the planned and implemented eradication activities, as well as the regional objectives achieved and those to be reached. All critical issues were analysed, and solutions were suggested.

## 3. Results

### 3.1. Italian Epidemiological Data

The data extracted from VETINFO confirm that Italy, following Commission Implementing Decision (EU) 2017/1910 [29], is able to maintain its official EBL-free status, although several persistent clusters of infection are still present in central and southern Italy.

In Table 1, the number and distribution of EBL Italian outbreaks recorded from 1 January 2018 to 2 July 2021 in VETINFO [33] is reported (data extraction 7 July 2021). An overall annual decrease was noted from 2018 to 2021, except in 2020, where 10 outbreaks were reported in central Italy.

From 2018 to 2021, the prevalence of EBL outbreaks remained under the threshold of 0.2% of controlled herds, as established by the regulatory criteria (Table 2).

**Figure 3:**
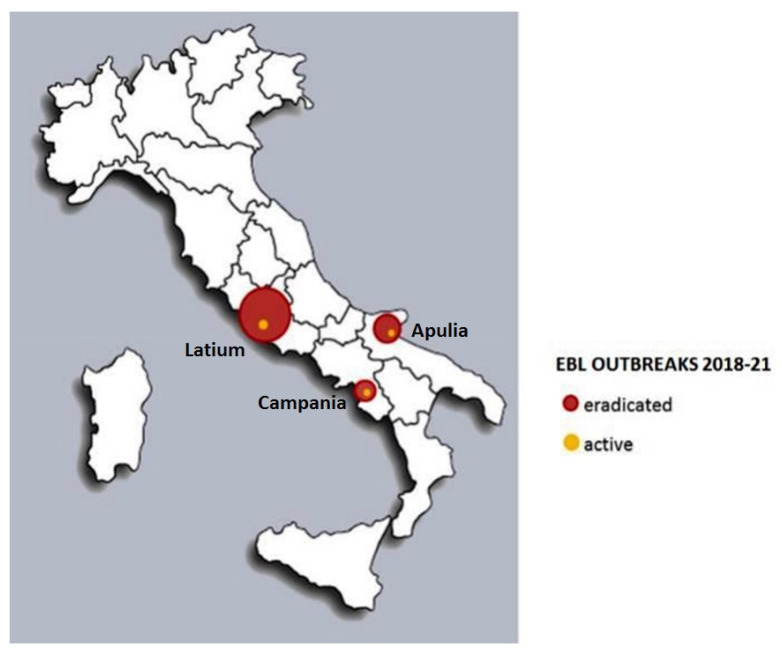
Notified EBL outbreaks in Italy, 2018–2021 (SIMAN, updated to 2 July 2021).

Figure 3 shows the evolution of EBL outbreaks in Italy recorded in SIMAN from 2018 to 2021 and highlights how the clusters of infection are concentrated in specific areas: Apulia, Campania, and Latium.

So far, in 2021, two active outbreaks have occurred in Latium, one in Apulia and one in Campania; indeed, in 2021, the registered prevalence of EBL outbreaks is 0.02% (Table 2). The outbreak in Campania, situated in the Benevento Province (Amorosi municipality), was recently discovered (July 2021), promptly notified in SIMAN, and managed according to the regulatory approach.

**Figure 4:**
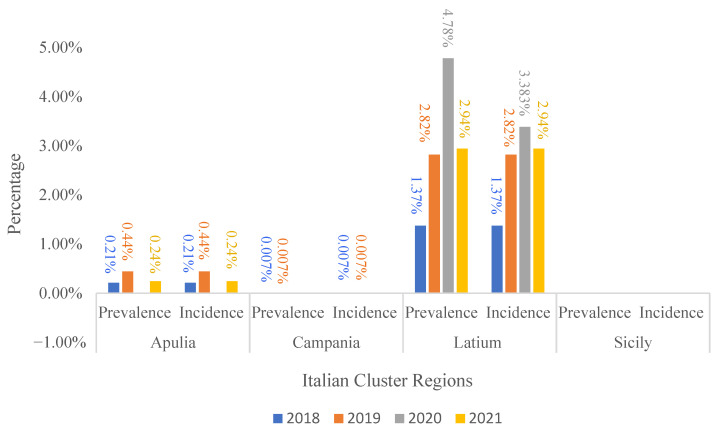
Outbreak prevalence and incidence in Italian clusters from 2018 to 2021 [33].

The prevalence and incidence of outbreaks in Italian clusters from 2018 to 2021 are shown in Figure 4. The eradication activity was effectively implemented by official local veterinary services, allowing a drastic reduction in EBL outbreaks in the Apulia, Campania, and Sicily regions during 2020–2021. Thus, the decreasing trend in EBL prevalence in these regions is largely attributable to control measures.

Sicily was found to have the lowest prevalence and incidence values; in fact, it obtained EBL-free status in March 2021. Latium still shows a constant and high infection prevalence and incidence, mainly due to the lack of cattle registration and the promiscuous breeding of herds in some municipalities. For Campania and Apulia, the prevalence and incidence data were distributed between 0.007% and 0.44%.

### 3.2. Data of the National Animal Register

The official data regarding the National Animal Register, as well as those regarding the surveillance activities, were extracted from the VETINFO platform, which includes all the information systems in support of the Italian public veterinary services. By using the special “Dashboard” tool dedicated to EBL surveillance and eradication plans, data for each cluster were collected about farm and animal monitoring, timing of outbreak settlement, transhumance and grazing management, and establishment of assembly operations (EAO) check.

CEREL, the Ministry of Health, the regions, and the official veterinary authorities used these data, associated with a gap analysis exercise, to perform risk assessments, and to plan corrective measures in the areas indicated.

In particular, in Campania, from 2018 to 2021, between 84% and 100% of the control activities in the EAO were performed. Conversely, in Apulia and Latium, during the same period, no checks in EAO were performed. Since 2021, Sicily has been excluded from this analysis because it is EBL free throughout the entire regional territory (Table 3).

**Figure 5:**
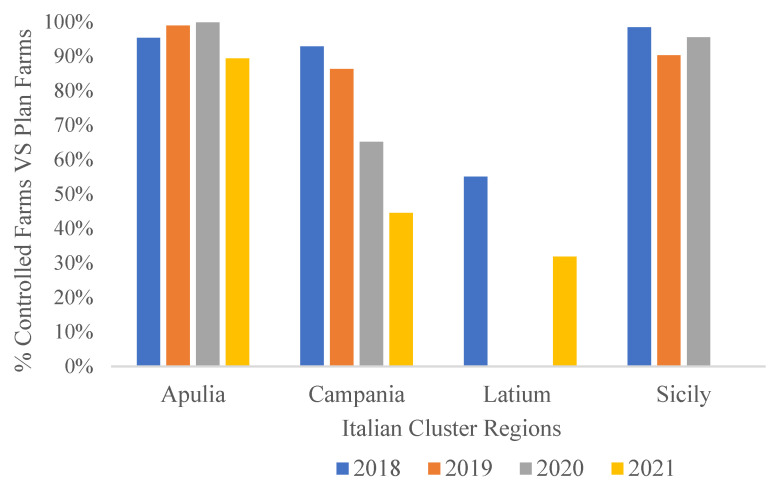
Percentage of controllable farms that were actually controlled in persistent clusters from 2018 to 2021 [33].

Figure 5 shows the percentage of controllable farms that were actually controlled in Italian clusters from 2018 to 2021. Apulia and Sicily had the best compliance rate, while Campania reported lower values. Data are not available for Latium in 2019–2020. Data were not reported for Sicily in 2021.

**Figure 6:**
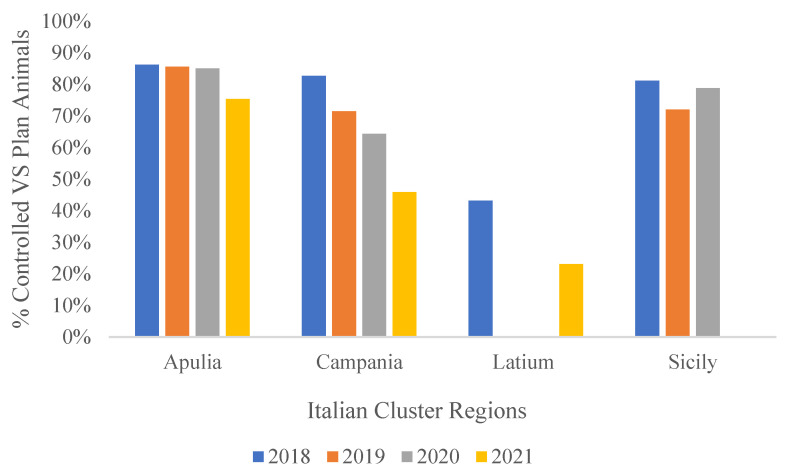
Percentage of controlled vs. planned to be controlled animals in persistent clusters from 2018 to 2021 [33].

Figure 6 shows similar data regarding controlled animals as a percentage of planned controls.

From 2018 to 2021, the prevalence of EBL-seropositive animals in farms remained below 0.004% of the controlled animals (Table 4).

### 3.3. Gap Analysis Results

All four regions containing EBL clusters were invited to participate in the gap analysis exercise, but Sicily was later exempted, having already demonstrated the eradication of the cluster identified in the Messina province. Later, Latium was excluded from the assignment, as in the gap analysis exercise, most objectives were not achieved, and regional data were considered too incomplete (Table 5).

Campania and Apulia promptly identified the infection areas within their territories and carried out adequate risk analysis. The questionnaire was partially filled out in only a few cases, without compromising the effectiveness of the evaluation (Table 6, Table 7 and Table 8). In these specific areas, where infection is still persistent, the gap analysis exercise confirmed that some factors are responsible for delaying the eradication process: free ranging animals, promiscuous breeding of herds, lack of collaboration between breeders, unrecorded animal movements, and lack of systematic documentation.

## 4. Discussion

The EBL eradication process, which began compulsorily in 1996, was completed more quickly in the regions of northern Italy due to favourable conditions, including, for example, the type of farming. In contrast, in the zootechnical context of central and southern Italy, the eradication process took longer, and took place after the implementation of the zootechnical register had been adequately completed and specific measures were adopted to eliminate some pockets of persistence of the virus.

In 2017, surveillance made it possible to achieve the requirements set by EU legislation to acquire EBL-free status throughout the country, despite the identification of four clusters in as many regions (Latium, Apulia, Campania, and Sicily). In these regions, serological tests were performed to control the spread of the infection and to remove infected animals. The ELISA test is the first tool for BLV screening programmes and follow-up studies, as it offers a high degree of sensitivity, while the AGID test guarantees a high degree of specificity, in accordance with the World Organisation for Animal Health [2].

The PCR assay from CEREL is used in addition to serological tests, mainly by testing seropositive animals slaughtered following EBL confirmation.

Despite the results achieved, the Ministry of Health’s commitment to the surveillance and control of the infection has not been reduced: new regulatory and technical instruments have, in fact, been applied with the aim of achieving complete eradication of the infection.

Through the adoption of even more stringent measures in the problem areas by the Ministerial Ordinances O.M. 28 May 2015 [32] and O.M. 23 June 2020 [37], greater efficiency in monitoring the activities and greater effectiveness in the pursuit of the planned objectives have been achieved, so much so that some regions, such as Sicily, have already reached EBL-free status.

For these reasons, Sicily was initially included as an Italian infection cluster, having had outbreaks of infection prior to 2018; subsequently, it was excluded from gap analysis. Indeed, in this region, the electronic identification and registration of animals has been strengthened, and more restrictive health measures have been established for the control of their movements, particularly for farms intended for transhumance, migration to summer pastures, and free-range breeding (temporary or permanent).

Good results have also been recorded in the Campania and Apulia clusters, which are in the process of being resolved. These regions have promptly identified some gaps in their surveillance systems and adopted the necessary corrective measures based on effective risk analysis. In particular, Campania implemented an eradication process based on constant evaluation of the epidemiological situation.

Although the specific health measures applied led to a drastic reduction of EBL outbreaks in Apulia, the program has not been able to eradicate the infection in some municipalities. In 2021, for example, there is still an active outbreak in the province of Foggia (municipality of San Marco in Lamis). This territory is characterised by difficult environmental, social, and economic conditions, and the activities of veterinary services are extremely difficult, also because of a lack of resources. In this persistent cluster, the circulation of BLV is favoured by conditions of promiscuity linked to free grazing and the inability to test the exposed population systematically and completely.

Unfortunately, the Latium region still lags behind in the solution of this problem. As in Apulia, some critical factors strictly closely rooted in the territory persist, despite having been known for some time. In this area, indocile or even feral cattle are reared, which makes it difficult to perform constant and complete checks on health parameters. Furthermore, the presence of unidentified animals not referable to any owner under conditions of promiscuity with reared cattle represents the most important reservoir of BLV. The removal of feral-like subjects is not easy and involves a series of problems that, at present, the local authorities have not been able to solve. Seropositive cases are frequently found in herds after years of negative results; the infection thus re-emerges after long periods of apparent freedom. In this context, farmers are often suspicious of the laboratory results and struggle to understand the epidemiological dynamics of a chronic disease that is almost always asymptomatic. Therefore, they do not always actively collaborate with the veterinary services in the application of controls.

For all of these reasons, as the application of eradication measures is difficult, the Latium region registered a high prevalence and incidence of infection during 2018–2021. The general failure to achieve the objectives in the gap analysis is due to the regional authority not carrying out the requested survey execution. Moreover, official data regarding some indicators (percentage of controllable farms and animals), useful for analysing the EBL control plan have still not been correctly incorporated into the digital system, and thus were not reported in this manuscript.

Beyond health considerations, delayed eradication has had serious economic consequences in these regions, both directly (e.g., cost of laboratory tests, of animal suppression, of farm inspections by the veterinary service, and other costs related to the obligations imposed by the restrictive measures in the clusters) and indirectly (e.g., movement blocks, reduced productive performance and economic depreciation of animals from non-free farms, increased consumer concerns regarding safety). In some cases, the pressure derived from the long-term application of restrictive measures has encouraged the illegal exchange and trade of animals, requiring police intervention.

It should also be remembered that, starting from 2011, the economic burden of the remediation activities of the EBL plan has been the sole responsibility of the Italian State, and is no longer co-financed by the EU.

After Italy achieved officially recognised EBL-free status, the Ministry of Health did not let its guard down, and, indeed, it implemented new systems to definitively achieve the objective of BLV eradication. In addition, the information systems supporting the activities of the surveillance plans were enhanced. The creation and use of the so-called “dashboard” has proved very useful in allowing real-time monitoring of the progress of health checks.

The Ministry of Health’s winning strategy in recent years has been to induce local authorities to adopt specific eradication measures, considering the specificities of the various areas concerned. In this context, an exercise based on “gap analysis” was proposed, in which the various regions had to analyse the progress of the eradication process, identify the critical issues that hinder the achievement of the goal, and identify possible ways of reducing these issues. This activity is a useful occasion for assessment that will hopefully bring tangible results in the near future.

Based on these assessments, some of the most important measures identified are the following:

- Improve the level of biosecurity of farms in order to reduce the risk of BLV circulation;
- Set up useful structures for the containment of livestock during the operations of identification and sampling in extensive farms;
- Adopt electronic identification tools (especially in populations reared in the wild);
- Remove all infection reservoirs, such as stray cattle, that cannot be traced back to any owner;
- Correctly supply the information systems and plan the necessary control actions in a targeted manner;
- Implement training and awareness programs for farmers.

During 2020 and 2021, the COVID-19 pandemic wave did not fail to affect these efforts. The activities of veterinary services have been severely affected and reduced due to health restrictions, with negative consequences on the EBL eradication process. It is hoped that veterinary services will be able to fully resume their activities as soon as possible. All the measures we have described require collaboration, commitment, and resources at different levels by all the actors involved (contact persons from the Ministry of Health, regions, CEREL, breeders, local administrations, public veterinary system), according to their respective skills. This is indispensable for accelerating the eradication process in the infected areas of our country. It is hoped that the use of these systems will accelerate progress towards the goal of complete eradication of BLV in the shortest possible time.

## Figures and Tables

**Table 1:** Number of EBL outbreaks notified by area, Italy 2018–2021 [33].

Year	Number of Outbreaks			
	Northern Italy	Central Italy	Southern Italy	Total
2018	0	5	5	10
2019	0	5	4	9
2020	0	10	1	11
2021	0	2	2	4
**Total**	**0**	**22**	**12**	**34**

**Table 2:** Prevalence of notified EBL outbreaks in controlled farms between 2018 and 2021 [33].

Years	No. Controlled Farms	No. Total Outbreaks	Prevalence of EBL Outbreaks
2018	30,458	10	0.03%
2019	24,649	9	0.03%
2020	20,812	11	0.05%
2021	14,796	4	0.02%

**Table 3:** EBL eradication plan surveillance activities in establishment for assembly operations resident in persistent clusters [33].

Years	Region	No. EAO under the Plan	No. Checked EAO	% EAO Coverage
2018	Apulia	Not done *	Not done	Not done
	Campania	12	12	100%
	Latium	1	0	0%
	Sicily	3	1	33.3%
2019	Apulia	Not done	Not done	Not done
	Campania	13	11	84.61%
	Latium	2	0	0%
	Sicily	1	1	100%
2020	Apulia	Not done	Not done	Not done
	Campania	11	10	90.9%
	Latium	2	0	0%
	Sicily	Not done	Not done	Not done
2021	Apulia	Not done	Not done	Not done
	Campania	11	11	100%
	Latium	2	0	0%
	Sicily	Not applicable	Not applicable	Not applicable

* Incomplete data.

**Table 4:** Prevalence of seropositive animals in farms between 2018 and 2021 [33].

Year	Animal Controlled	Seropositive Animals	Prevalence of EBL-Seropositive Animals
2018	894,442	41	0.004%
2019	685,601	18	0.002%
2020	524,254	18	0.003%
2021	355,391	8	0.002%

**Table 5:** Gap analysis of the application of the EBL eradication plan in the Latium infection cluster.

Sections	Current Epidemiological Situation	Desired Situation	Gaps between Current and Desired Situation	Measures to Be Applied	Necessary Resources	Risk Factors
Implementation of Eradication Process	C	C	C	C	C	C
Control of Local Area and Animal Farms	C	C	C	C	C	C
Control of Animals	C	C	C	C	C	C
Application of Planned Sanitary Measures	C	C	C	C	C	C
Effectiveness Checks	C	C	C	C	C	C

A—Objective completely achieved; B—Objective partially achieved; C—Objective not achieved.

**Table 6:** Gap analysis of the application of the EBL eradication plan in the Apulia infection cluster.

Sections	Current Epidemiological Situation	Desired Situation	Gaps between Current and Desired Situation	Measures to Be Applied	Necessary Resources	Risk Factors
Implementation of Eradication Process	A	B	B	A	B	A
Control of Local Area and Animal Farms	A	A	B	A	B	A
Control of Animals	A	A	A	A	A	A
Application of Planned Sanitary Measures	A	A	A	A	A	A
Effectiveness Checks	A	B	B	B	B	A

A—Objective completely achieved; B—Objective partially achieved; C—Objective not achieved.

**Table 7:** Gap analysis of the application of the EBL eradication plan in the Campania infection cluster.

Sections	Current Epidemiological Situation	Desired Situation	Gaps between Current and Desired Situation	Measures to Be Applied	Necessary Resources	Risk Factors
Implementation of Eradication Process	A	A	A	A	A	A
Control of Local Area and Animal Farms	A	A	A	A	B	A
Control of Animals	A	A	A	A	B	A
Application of Planned Sanitary Measures	A	A	A	A	A	A
Effectiveness Checks	A	A	A	A	A	A

A—Objective completely achieved; B—Objective partially achieved; C—Objective not achieved.

**Table 8:** General achievement of the gap analysis objectives in Italian infection clusters.

Italian Region	Sections				
	Implementation of Eradication Process	Control of Local Area and of Cattle/Buffalo Farms	Control of Animals	Application of Planned Sanitary Measures	Effectiveness Checks
**Apulia**	Partial analysis	Partial analysis	Gap detected	Partial analysis	Gap detected
**Campania**	Goal achieved	Goal achieved	Gap detected	Partial analysis	Goal achieved
Latium *	Not done	Not done	Not done	Not done	Not done

* Incomplete data.

## Data Availability

The data analysed in this study are available from the corresponding author upon reasonable request.

## References

[1] Hunter E., Casey J., Hahn B., Hayami M., Korber B., Kurth R., Neil J., Rethwilm A., Sonigo P., Stoye J., Van Regen-Mortel M.H.V., Fauquet C.M., Bishop D.H.L., Carstens E.B., Estes M.K., Lemon S.M., Maniloff J., Mayo M.A., McGeoch D.J., Pringle C.R. (2000). Family Retroviridae. Virus Taxonomy; Classification and Nomenclature of Viruses.

[2] OIE (2018). Enzootic bovine leucosis. Manual of Standards for Diagnostic Test and Vaccines.

[3] Rola M., Kuzmak J. (2002). The detection of bovine leukemia virus proviral DNA by PCR-ELISA. J. Virol. Methods.

[4] Feliziani F., Martucciello A., Iscaro C., Vecchio D., Petrini S., Grassi C., Bazzucchi M., De Carlo E. (2017). Bovine leukemia virus: Experimental infection in buffaloes and evaluation of diagnostic test reliability. Res. Vet. Sci..

[5] Molnar E., Molnar L., Guedes V.T.M., De Lima E.S.C. (2000). Naturally occurring bovine leukosis virus in water buffalo (*Bubalus bubalis*) in Brazil. Vet. Rec..

[6] Khalilian M., Hosseini S.M., Madadgar O. (2019). Bovine leukemia virus detected in the breast tissue and blood of Iranian women. Microb. Pathog..

[7] Hopkins S.G., Di Giacomo R.F. (1997). Natural transmission of bovine leukaemia virus in dairy and beef cattle. Vet. Clin. N. Am. Food Anim. Pract..

[8] Nuotio L., Rusanen H., Sihvonen L., Neuvonen E. (2003). Eradication of enzootic bovine leucosis from Finland. Prev. Vet. Med..

[9] Iscaro C., Felici A., Costarelli S., Dettori A., Maresca C., Feliziani F. (2014). Enzootic bovine leucosis in Italy: Update epidemiological situation and analysis of rules provided by the national eradication plan and the regional surveillance plans. Large Anim. Rev..

[10] Ooshiro M., Konnai S., Katagiri Y., Afuso M., Arakaki N., Tsuha O., Murata S., Ohashi K. (2013). Horizontal transmission of bovine leukemia virusfrom lymphocytotic cattle, and beneficial effects of insect vectorcontrol. Vet. Rec..

[11] Buxton B.A., Hinkle N.C., Schultz R.D. (1985). Role of insects in the transmission of bovine leukosis virus: Potential for transmission by stableflies, horn flies, and tabanids. Am. J. Vet. Res..

[12] Mekata H., Sekiguchi S., Konnai S., Kirino Y., Honkawa K., Nonaka N., Horii Y., Norimine J. (2015). Evaluation of the natural perinatal transmission of bovine leukaemia virus. Vet. Rec..

[13] Sajiki Y., Konnai S., Nishimori A., Okagawa T., Maekawa N., Goto S., Nagano M., Kohara J., Kitano N., Takahashi T. (2017). Intrauterine infection with bovine leukemia virus in pregnant dam with high viral load. J. Vet. Med. Sci..

[14] Marawan M.A., Mekata H., Hayashi T., Sekiguchi S., Kirino, Horii Y., Moustafa A.-M.M., Arnaout F.K., Galila E.S.M., Norimine J. (2017). Phylogenetic analysis of *env* gene of bovine leukemia virus strains spread in Miyazaki prefecture, Japan. J. Vet. Med. Sci..

[15] Mekata H., Yamamoto M., Hayashi T., Kirino Y., Sekiguchi S., Konnai S., Horii Y., Norimine J. (2018). Cattle with a low bovine leukemia virus proviral load are rarely an infectious source. Jpn. J. Vet. Res..

[16] Kettmann R., Burny A., Callebaut I., Droogmans L., Mammerickx M., Willems L., Portetelle D., Levy J. (1994). Bovine leukemia virus. The Retroviridae.

[17] Ghysdael J., Bruck C., Kettmann R., Burny A. (1984). Bovine Leukemia Virus. Curr. Top. Microb. Immunol..

[18] Feliziani F., Bazzucchi M., Giammarioli M., Casciari C., Iscaro C. (2018). Molecular characterization of Italian strains of bovine leukaemia virus. Proceedings of the 12th Annual Meeting of EPIZONE, ESVV.

[19] Acaite J., Tamosiunas V., Lukauskas K., Milius J., Pieskus J. (2007). The eradication experience of enzootic bovine leukosis from Lithuania. Prev. Vet. Med..

[20] Bartlett P.C., Sordillo L.M., Byrem T.M., Norby B., Grooms D.L., Swenson C.L., Zalucha J., Erskine R.J. (2014). Options for the control of bovine leukemia virus in dairy cattle. JAVMA.

[21] OIE Platform WAHIS. https://wahis.oie.int/#/dashboards/country-or-disease-dashboard.

[22] Commission Implementing Regulation (EU) 2021/620 of 15 April 2021 Laying Down Rules for the Application of Regulation (EU) 2016/429 of the European Parliament and of the Council as Regards the Approval of the Disease-Free and Non-Vaccination Status of Certain Member States or Zones or Compartments thereof as Regards Certain Listed Diseases and the Approval of Eradication Programmes for Those Listed Diseases. http://data.europa.eu/eli/reg_impl/2021/620/oj.

[23] Animal Disease Information System (ADIS). https://ec.europa.eu/food/animals/animal-diseases/animal-disease-information-system-adis_en.

[24] Council Directive 64/432/EEC of 26 June 1964 on Animal Health Problems Affecting intra-Community Trade in Bovine Animals and Swine. http://data.europa.eu/eli/dir/1964/432/oj.

[25] Decreto Ministeriale n. 358 del 02/05/1996 Regolamento Concernente il Piano Nazionale per L’eradicazione della Leucosi Bovina Enzootica. Gazzetta Ufficiale Serie Generale n. 160, 10/07/1996. http://www.oevcampania.it/wp-content/uploads/2017/04/Leucosi__D_M_358_96.pdf.

[26] Decreto Legislativo n. 196 del 22/05/1999 Attuazione della Direttiva 97/12/CE che Modifica e Aggiorna la Direttiva 64/432/CEE Relativa ai Problemi di Polizia Sanitaria in Materia di Scambi Intracomunitari di Animali delle Specie Bovina e Suina. Gazzetta Ufficiale n. 146, 24/06/1999—Supplemento Ordinario n. 120. https://www.camera.it/parlam/leggi/deleghe/testi/99196dl.htm.

[27] Commission Regulation (EU) 2016/429 of the European Parliament and of the Council of 9 March 2016 on Transmissible Animal Diseases and Amending and Repealing Certain Acts in the Area of Animal Health (‘Animal Health Law’). http://data.europa.eu/eli/reg/2016/429/oj.

[28] Commission Implementing Regulation (EU) 2020/2002 of 7 December 2020 Laying Down Rules for the Application of Regulation (EU) 2016/429 of the European Parliament and of the Council with Regard to Union Notification and Union Reporting of Listed Diseases, to Formats and Procedures for Submission and Reporting of Union Surveillance Programmes and of Eradication Programmes and for Application for Recognition of Disease-Free Status, and to the Computerised Information System. http://data.europa.eu/eli/reg_impl/2020/2002/oj.

[29] Commission Implementing Decision (EU) 2017/1910 of 17 October 2017 Amending Decision 93/52/EEC as Regards the Brucellosis (*B. melitensis*)-Free Status of Certain Regions of Spain, Decision 2003/467/EC as Regards the Official Bovine Brucellosis-Free Status of Cyprus and of Certain Regions of Spain, and as Regards the Official Enzootic-Bovine-Leucosis-Free Status of Italy, and Decision 2005/779/EC as Regards the Swine Vesicular Disease-Free Status of the Region of Campania of Italy. http://data.europa.eu/eli/dec_impl/2017/1910/oj.

[30] Iscaro C., Lomolino R., Palma D., Ruocco L., Possenti L., Feliziani F. Sistemi informativi per la sanità animale: Realizzazione di un nuovo strumento per il monitoraggio delle attività del piano di sorveglianza ed eradicazione della Leucosi Bovina Enzootica. Proceedings of the XIX Congresso Nazionale S.I.Di.L.V..

[31] Feliziani F., Lomolino R., Iscaro C., Costarelli S., Maresca C. (2018). L’Italia ha finalmente raggiunto i requisiti di indennità nei riguardi della Leucosi Bovina Enzootica. Large Anim. Rev..

[32] Ordinanza Ministeriale 28 Maggio 2015, Misure Straordinarie di Polizia Veterinaria in Materia di Tubercolosi, Brucellosi Bovina e Bufalina, Brucellosi Ovi-Caprina, Leucosi Bovina Enzootica. Gazzetta Ufficiale Serie Generale n.144, 24/06/2015. https://www.gazzettaufficiale.it/eli/id/2015/06/24/15A04879/sg.

[33] VETINFO. https://www.vetinfo.it/.

[34] Commission Delegated Regulation (EU) 2018/1629 of 25 July 2018 Amending the List of Diseases Set Out in Annex II to Regulation (EU) 2016/429 of the European Parliament and of the Council on Transmissible Animal Diseases and Amending and Repealing Certain Acts in the Area of Animal Health (‘Animal Health Law’). http://data.europa.eu/eli/reg_del/2018/1629/oj.

[35] Commission Implementing Regulation (EU) 2018/1882 of 3 December 2018 on the Application of Certain Disease Prevention and Control Rules to Categories of Listed Diseases and Establishing a List of Species and Groups of Species Posing a Considerable Risk for the Spread of Those Listed Diseases. http://data.europa.eu/eli/reg_impl/2018/1882/oj.

[36] Commission Delegated Regulation (EU) 2020/689 of 17 December 2019 Supplementing Regulation (EU) 2016/429 of the European Parliament and of the Council as Regards Rules for Surveillance, Eradication Programmes, and Disease-Free Status for Certain Listed and Emerging Diseases. http://data.europa.eu/eli/reg_del/2020/689/oj.

[37] Ordinanza Ministeriale 23 Giugno 2020 Proroga con Modifiche Dell’ordinanza del 28 Maggio 2015 e Successive Modificazioni, Recante: «Misure Straordinarie di Polizia Veterinaria in Materia di Tubercolosi, Brucellosi Bovina e Bufalina, Brucellosi Ovi-Caprina, Leucosi Bovina Enzootica». Gazzetta Ufficiale Serie Generale n.169, 07/07/2020. https://www.gazzettaufficiale.it/eli/id/2021/07/07/21A04133/SG.

[38] Tamba M., Pallante I., Petrini S., Feliziani F., Iscaro C., Arrigoni N., Di Sabatino D., Barberio A., Cibin V., Santi A. (2021). Overview of Control Programs for EU Non-regulated Cattle. Front. Vet. Sci..

